# Original Encounter with Antigen Determines Antigen-Presenting Cell Imprinting of the Quality of the Immune Response in Mice

**DOI:** 10.1371/journal.pone.0008159

**Published:** 2009-12-07

**Authors:** Valérie Abadie, Olivia Bonduelle, Darragh Duffy, Christophe Parizot, Bernard Verrier, Béhazine Combadière

**Affiliations:** 1 Institut National de la Santé et de la Recherche Médicale (INSERM) U945, Paris, France; 2 University of Pierre and Marie Curie (UPMC)- Univ Paris 06, Paris, France; 3 Assistance-Publique/Hopitaux-de-Paris, Immunity and Infection, Paris, France; 4 Institut de Biologie et Chimie des Protéines, UMR 5086 CNRS/UCBL, Lyon, France; New York University School of Medicine, United States of America

## Abstract

**Background:**

Obtaining a certain multi-functionality of cellular immunity for the control of infectious diseases is a burning question in immunology and in vaccine design. Early events, including antigen shuttling to secondary lymphoid organs and recruitment of innate immune cells for adaptive immune response, determine host responsiveness to antigens. However, the sequence of these events and their impact on the quality of the immune response remain to be elucidated. Here, we chose to study Modified Vaccinia virus Ankara (MVA) which is now replacing live Smallpox vaccines and is proposed as an attenuated vector for vaccination strategies against infectious diseases.

**Methodology/Principal findings:**

We analyzed *in vivo* mechanisms triggered following intradermal (i.d.) and intramuscular (i.m.) Modified Vaccinia virus Ankara (MVA) administration. We demonstrated significant differences in the antigen shuttling to lymphoid organs by macrophages (MΦs), myeloid dendritic cells (DCs), and neutrophils (PMNs). MVA i.d. administration resulted in better antigen distribution and more sustained antigen-presenting cells (APCs) recruitment into draining lymph nodes than with i.m. administration. These APCs, which comprise both DCs and MΦs, were differentially involved in T cell priming and shaped remarkably the quality of cytokine-producing virus-specific T cells according to the entry route of MVA.

**Conclusions/Significance:**

This study improves our understanding of the mechanisms of antigen delivery and their consequences on the quality of immune responses and provides new insights for vaccine development.

## Introduction

The *in vivo* mechanisms triggered by antigen delivery have been studied much less than those related to T and B-cell and protective immunity. In particular, the processes of local uptake and transport of antigen, the status of antigen-presenting cells (APCs), and the circumstances of T cell priming remain elusive.

Modified Vaccinia virus Ankara (MVA) is now replacing live Smallpox vaccines that were successfully used during decades without any knowledge of mechanism-inducing immune responses [1], [2], [3], [4], [5], [6]. MVA is an attenuated strain of Vaccinia virus (VV) lacking the ability to replicate *in vitro* and *in vivo* [7], [8], [9], [10] and is currently being investigated as a candidate recombinant vaccine for infectious diseases [11]. The efficacy of MVA immunization has been investigated in several animal models [1], [3], [4], [6] however, without rigorous comparison of distinct routes of immunization. The use of conventional intramuscular (i.m.) and intradermal (i.d.) delivery routes for immunization remains part of the intense debate. We investigated whether the site of antigen delivery may dictate the differential participation of APCs in the initial steps of T cell priming, activation and quality of cellular immune responses. Deciphering the mechanisms involved in T cell mediated immunity is of particular interest for MVA, a leading option for the development of HIV vaccines and tumor immunotherapy [12].

The complex relationships linking APC identity, recruitment, antigen-loading, and localization, to the intensity and quality of immune responses make it necessary to analyze the early steps of T cell priming in greater detail. Human studies have highlighted the role of IFN-γ-producing CD4 Th1 cells in the generation of long-term cell memory to VV [13], [14], for roughly half of long-term vaccinees specifically lose their VV-specific CD8 T cell response [14]. In addition, recent studies in humans vaccinated with VV showed a major amplification of CD8 cells at priming [15]. These studies have focused on the magnitude of the effector T cell response, while recent works have underlined the importance of defining distinct functional T cell populations that reflect the quality of the T cell response [16]. Route-dependent effects of MVA have been investigated for the localization of CD8 T cell priming [17] and for antigen processing and MHC-Class I presentation [18]. It has also been shown that the immunization route may profoundly affect the recognition of MHC-Class I restricted poxvirus epitopes [19]. According to the literature, MVA has a pronounced tropism for monocytes (Mos)/macrophages (MΦs) but also infects dendritic cells (DCs) and B cells, all of which can act as professional APCs [20], [21]. In a model of VV subcutaneous (s.c.) administration, clustering was observed only between VV-specific CD8^+^ T cells and infected DCs, although most VV-infected cells in draining lymph nodes (DLNs) were MΦs [22], [23]. The direct involvement in T cell priming of other APCs, such as MΦs, has so far not been demonstrated.

In this study, we investigated the early mechanisms of MVA–induced immune response after i.d. and i.m. immunization. Our findings demonstrate that i.d. and i.m. administration target different APCs that differentially shape the virus-specific cell-mediated immune response of both CD4 and CD8 cells. The accessibility of MVA antigen to different APCs at the site of immunization dictates the occurrence and extent of cellular immunity.

## Results

### The site of antigen delivery shapes both the intensity and the quality of MVA–induced cellular immune response

**Figure 1:**
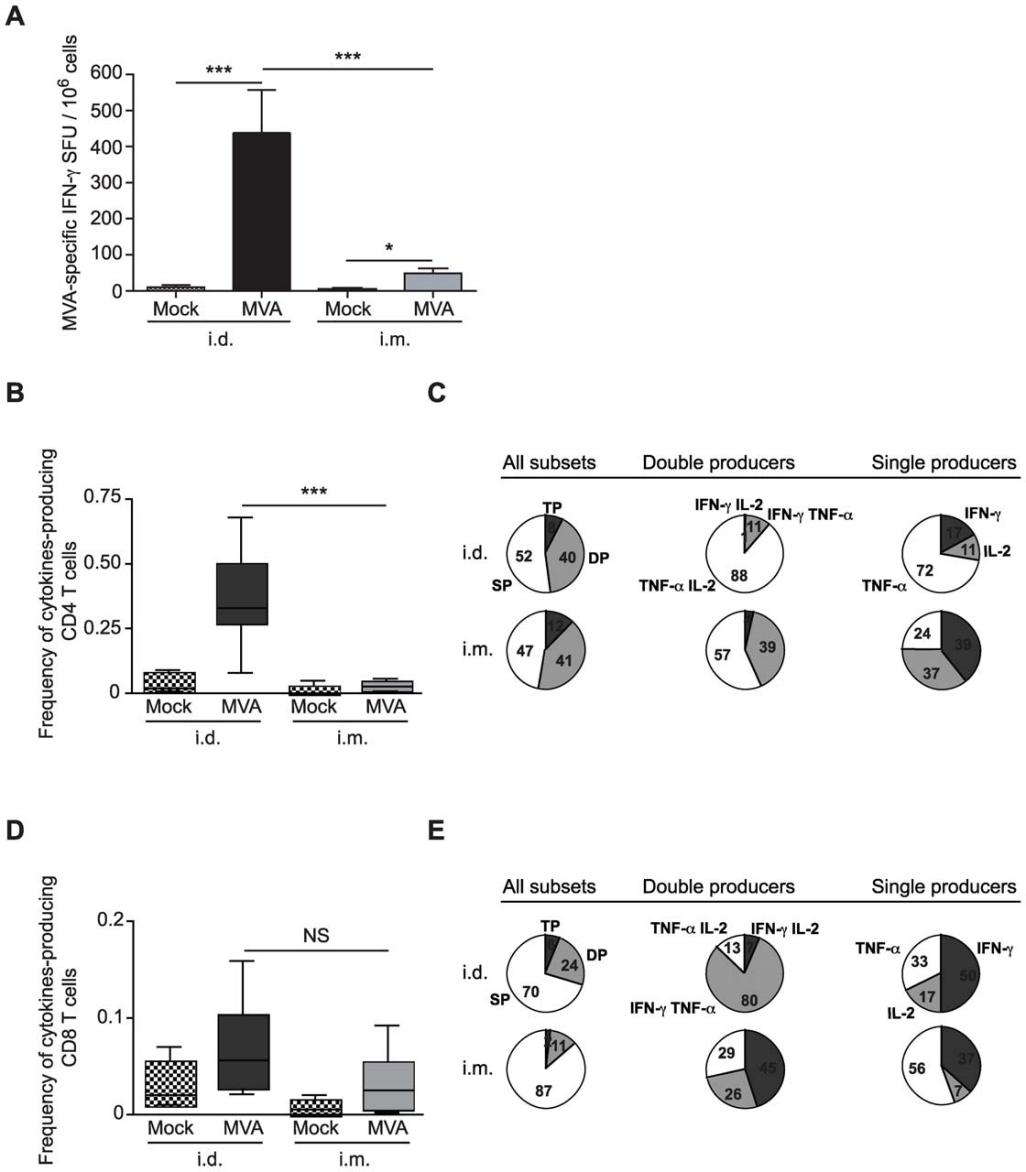
Magnitude and quality of MVA–specific T cell responses following i.d. and i.m. antigen delivery. (A) C57BL/6 mice were immunized i.d. or i.m. with 5.10^6^ PFU of MVA or saline buffer as a control. Seven days after inoculation, IFN-γ–producing DLNs T cells were quantified by ELISPOT assay. (B) Mice received 5.10^6^ PFU of MVA i.d. or i.m.. MVA–specific T cell responses in the DLNs on day 7 were measured with an intracellular cytokine staining assay. The magnitude of MVA–specific CD4^+^ T cells producing cytokines is shown. (C) The functional composition of the CD4^+^ T cell response is presented. The pie charts present the mean frequencies of the CD4^+^ T cells positive for the indicated cytokines. TP, triple producers; DP, double producers; and SP, single producers. (D) The magnitude of the total CD8^+^ T cell response is presented. (E) The cytokine coexpression profile of MVA–specific CD8^+^ T cells is shown (as in C). * p<0.05, *** p<0.0001. Data are representative of two (B–E) or three (A) independent experiments (n = 10 individual mice for each group).

Despite numerous comparisons of antigen delivery routes for the induction of immune responses against VV, there appears to be no comparisons between the standard i.d. and i.m. routes of immunization. We thus compared both the intensity and quality of cellular (Figure 1) and humoral (Figure S1) immune responses after MVA immunization by these routes. MVA–specific T cell responses in LNs draining the muscle (the inguinal DLNs, IDLNs) was much lower than the MVA–specific T cell response in LNs draining the skin (auricular draining lymph nodes, ADLNs) as assessed by an IFN-γ production ELISPOT (Figure 1A). Similar results were observed with MVA encoding for HIV-1-reverse transcriptase (rMVA–RT) (Figure S2).

To determine whether the MVA delivery route induced distinct polyfunctional effector T cells, DLN cells were harvested seven days after MVA delivery at the peak of effector T cell responses (Figure S3). Simultaneous expression of IFN-γ, TNF-α, and IL-2 by MVA–specific T cells was analyzed with flow cytometry and the Boolean gating function of FlowJo software (Figure 1B–1E, and Figure S4). Of the various T cell functions, secretion of IFN-γ, TNF-α, and/or IL-2 is particularly relevant for virus-specific T cell response studies [16]. As expected, the frequency of total cytokine-producing CD4^+^ T cells was significantly higher in the ADLNs of mice that received MVA i.d. than in IDLNs from mice immunized by the i.m. route (Figure 1B). However, the frequency of cytokine-producing CD8^+^ T cells was similar in both groups, regardless of the route of inoculation (Figure 1D). Remarkably, we noted significant differences in the multifunctionality of these induced T cells according to the site of antigen entry (Figure 1C and 1E). Pie charts analyses summarize differences in quality of cytokine producing CD4 and CD8 cells after i.d. and i.m. immunization. In the group with MVA administered i.d., the MVA–specific CD4^+^ T cells producing TNF-α were significantly higher compared to the MVA administered i.m. group (Figure 1C). Strikingly, the total cytokine response of CD8^+^ T cells was sharply different according to the MVA administration route. The frequency of multiple cytokine-producing CD8^+^ T cells was higher after i.d. administration, and IFN-γ-TNF-α production dominated the response (Figure 1E).

The magnitude of MVA–specific T cell response was also higher in the spleen of mice that received MVA i.d. compared to those administered i.m., and differences in the multifunctionality of MVA–specific T cells were observed according to the site of antigen entry (Figure S4).

Thus, it is not only the magnitude of the T cell response that is affected but also its quality: the MVA delivery route determines distinct patterns of cytokine production by antigen-specific T cells.

### The persistence of MVA at the inoculation site induced prolonged and distinct innate cell recruitment

**Figure 2:**
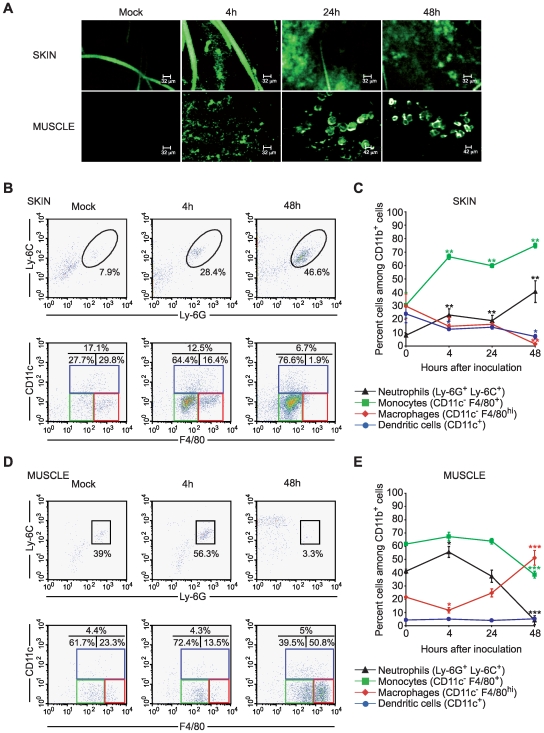
Persistence of rMVA–*egfp* particles and phenotype of cells infiltrating the inoculation sites after i.d. and i.m. antigen delivery. (A) Fibered confocal fluorescence microscopy of skin and muscle from 4 to 48 h after inoculation of 5.10^6^ PFU of rMVA–*egfp* or saline buffer as a control by the i.d. and i.m. routes. (B) Flow cytometric analyses of skin-infiltrating cells. Ly-6C^+^Ly-6G^+^ PMNs were gated on CD11b^hi^ cells. Mo/MΦ (F4/80^+^CD11c^−^) and DC (CD11c^+^) subpopulations were gated on CD11b^+^ cells. (C) Results are expressed as the percentage of CD11b^+^ cells isolated from ears at 4, 24, and 48 h after injection of 5.10^6^ PFU of MVA. PMNs are expressed as the percentage of CD11b^hi^. Values at point 0 correspond to the percentage of positive cells extracted from ears of control mice. Each percentage represents the mean values obtained for three mice per group. *P<0.05, **P<0.01. This experiment was performed twice with similar results. (D) Similar representative flow cytometric analyses of muscle infiltrating-cells. The percentages of cells for each subpopulation are indicated in the quadrant. (E) CD11b^+^ cells present in muscle were analyzed for expression of both Ly-6C and Ly-6G, and for expression of CD11c and F4/80 by flow cytometry. Results are expressed as the percentage of CD11b^+^ cells (except PMNs that are all CD11b^hi^) isolated from muscle at 4, 24, and 48 h after injection of 5.10^6^ PFU of MVA. Values at point 0 correspond to the percentage of positive cells extracted from control mice. Each percentage represents the mean values obtained for three mice per group. *P<0.05, **P<0.01, ***P<0.0001. This experiment was performed twice with similar results.

The intrinsic structure of skin and muscle and the difference in their ability to be infiltrated by innate immune cells may modulate antigen persistence and its conveyance to lymphoid organs. To determine if the discrepancy observed between antigen-specific T cell responses resulted directly from early innate events at the antigen delivery site, we next evaluated the distribution of MVA at the inoculation sites and subsequent innate cell recruitment after i.d. and i.m. inoculation of MVA recombinant for the enhanced green fluorescent protein (rMVA–*egfp*) (Figure 2).

As Figure 2A shows, localization of rMVA–*egfp* at inoculation sites was assessed at several time points using a fibered-confocal microscope (Cell Vizio®, Mauna Kea technologies) [24]; high amounts of MVA particles were clearly present in the mouse skin (upper panels) and muscle (lower panels) and persisted there for 4 to 48 h after both i.d. and i.m. antigen delivery.

We then evaluated the nature, kinetics, and behavior of cells both resident in and infiltrating skin and muscle at several time points (Figure 2B–2E). Analysis of cells isolated from the skin revealed a significant and sustained increase in CD11b^hi^CD11c^−^Ly-6G^+^Ly-6C^+^ polymorphonuclear neutrophils (PMNs) in the dermis from 4 to 48 h after i.d. MVA inoculation (Figure 2B, upper dot-plots, and Figure 2C). At the same time, the frequency of the subpopulation of CD11b^+^CD11c^−^F4/80^lo^ Mos increased strongly (Figure 2*B*, lower dot-plots, and Figure 2*C*). In the dermis, two cell subsets expressing F4/80 were also identified as CD11b^+^F4/80^hi^CD11c^−^ dermal MΦs and CD11b^+^F4/80^+^CD11c^+^ dermal DCs. The frequency of both fell at 4 and 48 h after i.d. MVA injection (Figure 2B, lower dot-plots, and Figure 2C).

After i.m. MVA delivery, however, we observed an early and transient increase in PMNs frequency at four h after inoculation that significantly fell at 48 h (Figure 2D, upper panels, and Figure 2E). In addition, no significant changes in CD11c^+^ DC frequency were observed. In contrast, the CD11c^−^F4/80^hi^ MΦ subset decreased slightly four h after i.m. antigen delivery and then increased continuously up to 48 h. This mirrored the behavior of the CD11c^−^ F4/80^+^ Mo subset that declined at 48 h in the muscle. Because both Mo/MΦ subsets expressed high levels of Ly-6C and CD11b, the variation observed may represent phenotypic changes due to Mos differentiation rather than renewal during cell infiltration of muscle tissue.

Collectively, these results suggest that the dynamics and kinetics of innate cell populations differs significantly in skin and muscle. In particular, although the initial frequencies of Mos and MΦs were similar at both inoculation sites, there were more DCs in the skin between 4 to 48 hours. The availability of APCs (i.e., DCs and MΦs) in the skin may be a critical factor in shuttling antigen to lymphoid tissues and in its subsequent presentation to T lymphocytes.

### Influence of the site of antigen delivery on the rate of antigen distribution toward DLNs

**Figure 3:**
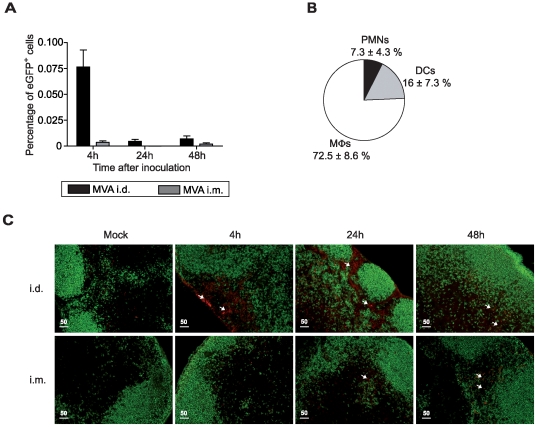
Differential biodistribution of rMVA–*egfp* particles after i.d. and i.m. antigen delivery. (A) At 4 to 48 h after i.d. and i.m. injection of 5.10^6^ PFU of rMVA–*egfp*, DLN suspensions were analyzed for the presence of eGFP^+^ cells by flow cytometry. Each histogram bar represents the mean percentage for three independent experiments (n = 3 mice per group and per time point for each experiment). (B) Four h after rMVA–*egfp* inoculation, eGFP^+^ ADLN cells were analyzed by flow cytometry and identified as CD11b^+^F4/80^+^ M√s, CD11b^+^CD11c^+^ mDCs, and Ly-6G^+^Ly-6C^+^ PMNs. Pie chart represents the proportion of each eGFP^+^ cell subset for three independent experiments (n = 3 mice per group and per time point for each experiment). (C) Immunohistochemistry of DLN samples stained with anti-B220 (green) and anti-vaccinia (red) to localize MVA–infected cells in the node. Slides were analyzed under an Olympus fluorescence microscope with a UPlanFL N 10×/0.30 lens.

We further assessed the kinetics of the distribution of rMVA–*egfp* in DLNs and quantified the eGFP^+^ cells by flow cytometry (Figure 3A). The number of GFP-expressing cells peaked in ADLNs (after i.d. immunization) as early as four h after injection, at ≈4000 per ADLN (Figure 3A). This number then declined rapidly with only ≈300 positive cells detected at 24 h and 48 h after i.d. inoculation of rMVA–*egfp*. eGFP^+^ particles were detected in 72.5±8.6% of MΦs (CD11b^+^F4/80^hi^CD11c^−^), in 16±7.3% of myeloid DCs (mDCs, CD11c^+^CD11b^+^) and 7.3±4.3% of PMNs (Figure 3B). These results show that rMVA–*egfp* particles were associated with myeloid-derived cells in ADLNs within the first four h of i.d. antigen delivery. Extremely low amounts of GFP-expressing cells were observed in the IDLNs after i.m. injection (Figure 3A); there were around 200 eGFP^+^ cells at four h afterwards. This paucity of GFP-expressing cells prevented us from performing further analysis after i.m. injection.

We further assessed the presence of viral antigens in DLNs, using an anti-vaccinia antibody for histological analyses (Figure 3C). After i.d. injection, MVA proteins were detected as early as four h afterwards in the ADLN subcapsular sinus, but also at 24 and 48 h in deeper areas beneath and between B cell follicles (Figure 3C, upper panels). In contrast, after i.m. injection, low levels of viral proteins were detected at later time points (24 and 48 h) in the T cell area (Figure 3C, lower panels).

Our results show for the first time major differences between the i.d. and i.m. routes for innate cell infiltration, virus shuttling, and antigenic distribution. Whereas i.d. inoculation leads to rapid transfer of MVA into the ADLNs with viral antigen persisting for at least the first 48 h after immunization, i.m. inoculation of MVA induces only slight dissemination of MVA particles at late time points.

### Influence of the site of antigen delivery on the LN cellular microenvironment and subsequent cell recruitment

**Figure 4:**
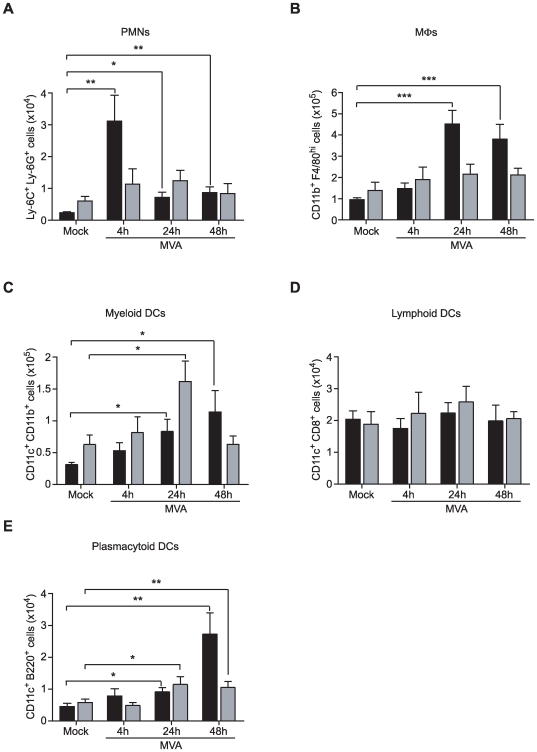
Cell recruitment into DLNs according to route of MVA administration. At 4 to 48 h after i.d. (black bars) or i.m. (grey bars) MVA inoculation, leukocyte subpopulations present in DLN suspensions were characterized by flow cytometry (A, PMNs; B MΦs; C mDCs; D lymphoid DCs; E pDCs). Each histogram bar represents the mean percentage for three independent experiments (n = 3 mice per group and per time point for each experiment). *P<0.05, **P<0.01, ***P<0.0001.

We next investigated cellular heterogeneity of innate cell recruitment and DC populations in DLNs where immune response begins. Indeed, others have shown that the accumulation of APCs after initial antigen encounter might be linked with the initiation of adaptive immune responses [25], [26]. At several time points after i.d. and i.m. antigen delivery, DLNs were isolated for flow cytometric identification of cell subpopulations, including Mos, MΦs, PMNs, and DCs (Figure 4A–4E). The PMN subset multiplied by 13 in the ADLNs within four h of the i.d. MVA inoculation, compared with control mice, and remained three to four times higher at 24 and 48 h (Figure 4A). In contrast, after i.m. immunization, PMN levels in IDLNs did not change significantly (Figure 4A). This sharp contrast suggests differences in initial inflammation by i.d. and i.m. routes. In addition, increased amounts of CD11b^+^F4/80^hi^ MΦs were detected at 24 and 48 h after i.d. but not i.m. inoculation (Figure 4B). In contrast, CD11b^+^CD11c^+^ mDCs quantitatively increased from 24 to 48 h after both i.d. and i.m. injections compared with their respective control mice (Figure 4C). In addition, the number of CD11c^+^CD8^+^ lymphoid DCs remained constant both in ADLNs and IDLNs (Figure 4D) and CD11c^+^B220^+^ plasmacytoid DCs (pDCs) increased at 24 and 48 h after MVA inoculation by both routes (Figure 4E).

Thus, both i.d. and i.m. antigen delivery produced remarkable changes in myeloid and innate cell populations in the respective DLN suggesting that the initial influxes of leukocytes in DLNs may play a role in the intensity and quality of adaptive immune response to MVA antigens.

### Differential APC involvement in T cell priming according to the original antigen delivery site

**Figure 5:**
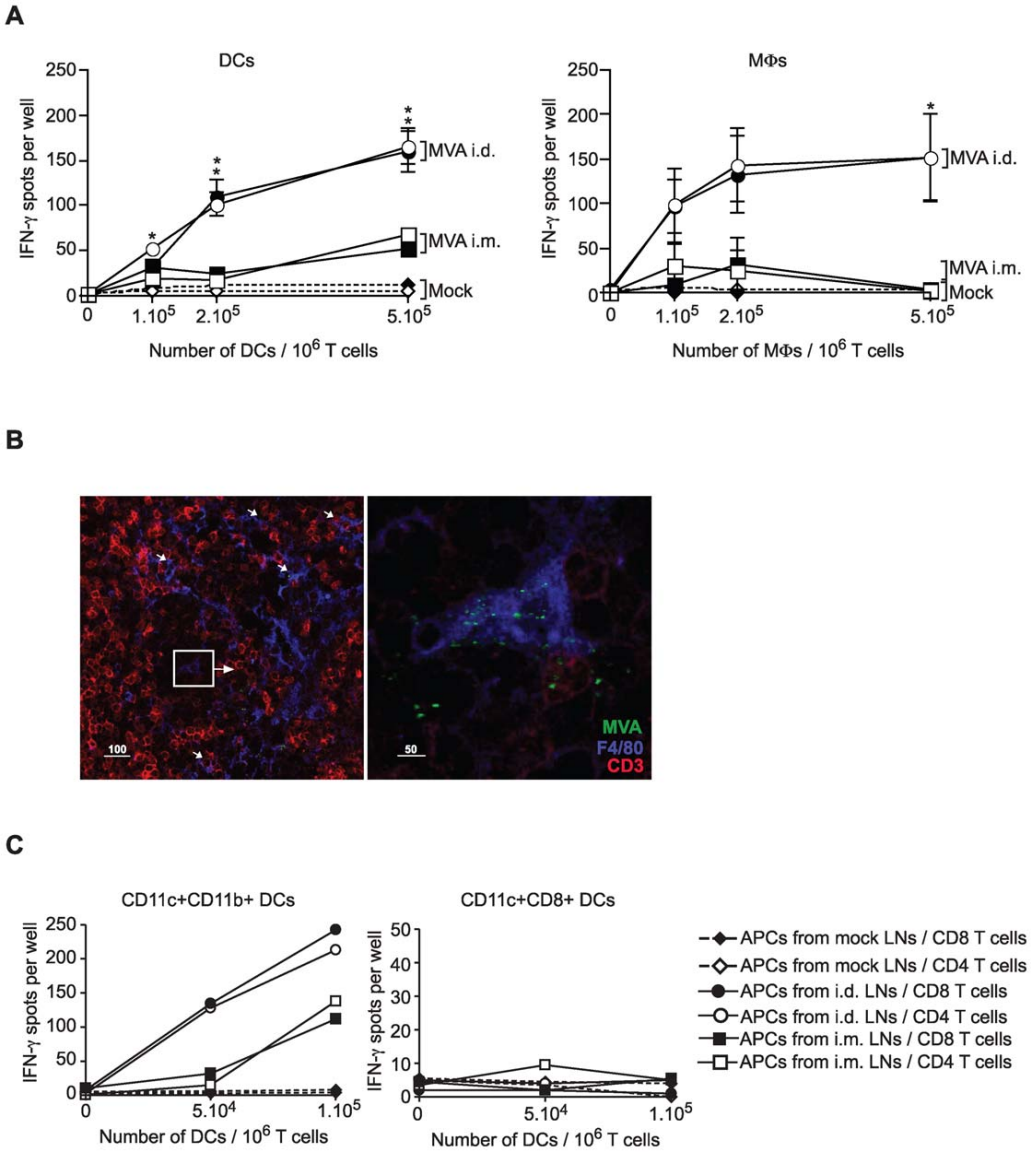
Differential involvement of myeloid cells in T cell priming according to antigen delivery route. (A) Number of IFN-γ positive spots produced by CD4^+^ (open symbols) and CD8^+^ (black symbols) T cells after incubation with the indicated number of DCs (left panel) or MΦs (right panel) isolated from Mock (⋄, ♦), ID (○, •), and IM (□, ▪) MVA–inoculated DLNs on day 2. Each data point represents triplicates from three independent experiments. (B) 48 h after MVA i.d. administration, a section cut from frozen ADLN was immunostained with anti-F4/80 (blue), anti-CD3 (red), and anti-vaccinia (green) to localize interactions between MVA–infected F4/80^+^cells and T cells in the node. Slides were analyzed under a Leica Sp2 AOBS confocal microscope with a 63x/1.4 NA lens. (C) CD11c^+^ DCs from DLNs on day 2 were sorted into CD8α^+^ DCs and CD11b^+^ DCs. DC subsets were cocultured with CD4^+^ (open symbols) and CD8^+^ (black symbols) naïve T cells, and tested in duplicate wells for their ability to induce IFN-γ production by T cells. Data are representative of three independent experiments.

Because lymphoid organs are settled by successive and different waves of innate cells likely to be involved in antigen capture and presentation to T lymphocytes, we examined whether the accessibility of MVA antigen to different APC subpopulations dictates the occurrence and extent of cellular immunity. We isolated CD11c^+^ DCs and CD11c^−^F480^+^ MΦs (Figure S5A) and tested their ability to stimulate IFN-γ production by naive CD4 and CD8 T lymphocytes in a 36-hour IFNγ-ELISPOT assay (Figure 5). First, both DCs and MΦs isolated from ADLNs after i.d. immunization induced much stronger MVA–specific effector CD4^+^ and CD8^+^ T cells than did those isolated from IDLNs after i.m. immunization (Figure 5*A*) confirming superior intensity of cellular responses after i.d. immunization. The capacity of these cells to interact with T cells was visualized in ADLNs 48 h after i.d. MVA inoculation using confocal microscopy (Figure 5B).

Because lymphoid and myeloid DCs have been a matter of debate in T cell priming, we next purified CD11b^+^CD11c^+^ myeloid and CD8α^+^CD11c^+^ lymphoid DCs to test their ability to prime T cells. Surprisingly, mDCs induced IFN-γ production by CD4^+^ and CD8^+^ T cells more potently than lymphoid DCs (Figure 5C).

These data demonstrate that APCs of myeloid origin are involved in T cell priming after MVA inoculation. i.d. antigen delivery mobilizes two distinct APC subsets, both mDCs and MΦs, whereas i.m. inoculation appears to involve only the former.

### Intradermal antigen delivery-dependent myeloid APCs imprinting of MVA–specific T cell responses

**Figure 6:**
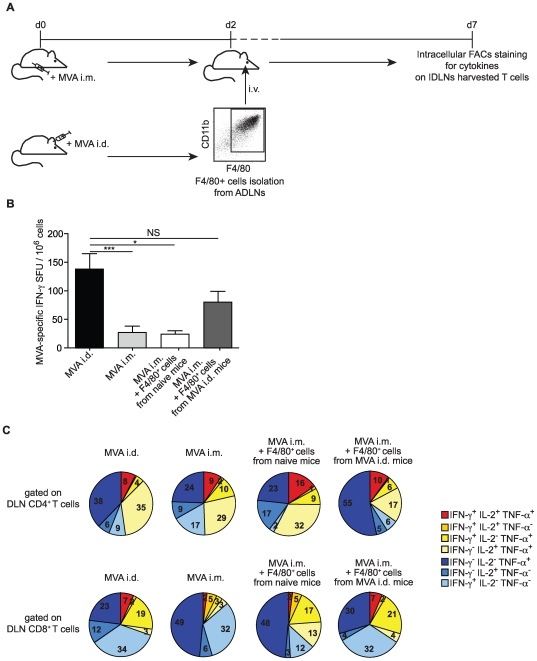
Myeloid APCs qualitatively shape MVA–specific T cell responses. (A) F4/80^+^ cells were isolated from MVA i.d. inoculated mice on day 2 or from control mice, and transferred intravenously into i.m. MVA–inoculated mice the same day. DLNs were isolated from recipient mice on day 7 to determine the cytokine coexpression profile of T cells. (B) Mice were inoculated with MVA i.d. or i.m. and received intraveneously F4/80^+^ cells isolated from MVA i.d. inoculated mice on day 2 or from control mice. Seven days after inoculation, IFN-γ–producing DLNs T cells were quantified by ELISPOT assay. Representative data from two independent experiments are presented (n = 10 individual mice for each group). * p<0.05, *** p<0.0001, NS Non Significant. (C) DLNs from mice inoculated with MVA i.d. or i.m. and from MVA i.m. immunized mice that received F4/80^+^ cells were analyzed on day 7 for their MVA–specific T cell response. DLN cells were stimulated *in vitro* for 12 h with MVA antigen. Cell surfaces were stained with anti-CD8α and anti-CD4, and then cells were intracellularly stained with anti-IFN-γ, anti-TNF-α, and anti-IL-2. Cytokine co-expression patterns are represented as the mean frequencies of the T cell subsets positive for the indicated cytokines. Representative data from two independent experiments are presented (n = 10 individual mice for each group).

Although the main function of APCs is to trigger T cells into cell cycle progression, it is now well established that functionally distinct APC subsets can also influence the subsequent development of these dividing cells and their aptitude to secrete different patterns of cytokines [27]. We next tested whether the quality of APCs can directly influence the quality of the T cell responses. We found in our model that the most powerful DCs that stimulate T cells were also positive for the Ly-6C and F4/80 markers and expressed MHC II strongly (Figure S5B). Purified F4/80^+^ cells (mDCs and MΦs) isolated from control or i.d. MVA injected mice at day 2 post-immunization were then i.v. injected into recipient mice that had received MVA by the i.m. route (Figure 6A). We analyzed the intensity of T cell effector immune response by measuring IFN-γ production and we characterized the cytokine production patterns on day 7, by the multiparametric flow cytometric analysis in different groups described in Figure 6.

Interestingly, transferring F4/80^+^ cells isolated from i.d. injected mice, but not naive mice, increased the magnitude of MVA–specific T cell response (Figure 6B), and shaped the cytokine distribution with increased frequency of TNF-α−producing CD4^+^ T cells (Figure 6C, upper panel). Remarkably, the transfer of F4/80^+^ cells isolated from i.d. MVA injected mice, but not naive mice, to MVA i.m. inoculated mice restored the cytokine distribution of CD8^+^ T cells observed for the i.d. route (Figure 6C, lower panel). The analysis of the splenic compartment led to similar observations (Figure S6). Therefore, these results indicate that specific APCs originally imprinted at the site of immunization have the capacity to shape the quality of the immune response.

## Discussion

The results presented in this report provide for the first time a demonstration of the impact of the antigen delivery route on the quality of the immune response, a quality that reflects the functional status of effector T cells. Several studies have emphasized the influence of environmental cues, antigen dose, and specificity of the APCs recruited to the T cell activation site in shaping the response by modulating cell function (reviewed in [27]). Here, we provide *in vivo* evidence that the route of delivery affects all these factors that determine the type of immune response. In particular, we demonstrated that i.d. injection resulted in better antigen distribution and more sustained APC recruitment into DLNs than with i.m. injection. These APCs, which comprise both DCs and MΦs, are involved in T cell priming and are responsible for tuning the quality of the elicited immune response.

Many routes of immunization have been used to study poxvirus-induced immune responses in mice. However, we chose to compare the i.m. route, currently used in human vaccination trials to overcome untoward effects such as local skin reactions induced by s.c. or i.d. vaccine delivery, with the i.d. route, which closely mimics the original VV vaccination route of scarification. Strikingly, the mode of antigen delivery affected not only the strength but also the quality of the antigen-specific response, as measured by the total cytokine response.

The capacity of a viral vector such as MVA to elicit high-frequency immune responses depends critically on the efficient presentation of antigens to T lymphocytes. This may be particularly true for MVA, which has impaired replication ability. This observation led us to explore the capacity of the antigen to reach the secondary lymphoid organs and the identity of the immunocompetent cells recruited and potentially involved in T cell priming and subsequent T cell activation. Our data demonstrate that i.d. antigen delivery results in the rapid spread of the entire virus to DLNs in contrast to the i.m. antigen administration. The amount of antigen that reaches the DLN seems to be higher after i.d. compared to i.m. route, which could explain the intensity of the immune response of i.d. route compared to i.m. route. However both routes of immunization induced significantly intense immune responses, compared to control mice.

Anatomic constraints may promote or prevent the dissemination of peripheral antigenic compounds, for the vasculature in muscle is much less extensive that than in skin, which has a sprawling network of lymphatic vessels. After i.m. inoculation, we found GFP associated mainly with muscle fibers. This is consistent with recent observations showing that after i.m. inoculation of a recombinant strain of MVA expressing the luciferase reporter gene, the luciferase signal was restricted mainly to the muscle [28]. Alternatively, the discrepancy in antigen transport from the site of inoculation toward secondary lymphoid organs may reveal differences in the capacity of innate immune cells to be recruited within the tissue and subsequently migrate towards these organs. This may result in differential inflammatory environments.

We also observed sharply different behavior of incoming leukocytes according to whether the inoculation site was skin or muscle. After i.d. inoculation, a sustained increase in the number of PMNs and Mos recovered from the skin was observed, while the increase in PMNs in the muscle after i.m. inoculation was only transient. Interestingly, at early time points after i.d. administration, MVA moved to the ADLNs at the same time as a substantial influx of both PMNs and Mos/MΦs was also observed there. In view of previous studies demonstrating the role of Mos/MΦs and PMNs in bacteria transport from the periphery to DLNs [29], [30], the early burst of these cell populations at both the i.d. inoculation site and in the subcapsular sinus of DLNs suggests that these cells may have some role in shuttling the antigen. However, it is also possible that most of the viral particles may drain directly from the skin to ADLNs. In contrast, after i.m. antigen delivery, antigen positive cells were detected later into the deeper paracortex of DLNs.

MVA particles in ADLNs were found to be associated mainly with several cell populations of myeloid origin, specifically MΦs, mDCs, and PMNs. Despite its non-replicating and non-propagating properties, MVA presents the same pattern of *in vivo* viral tropism as previously observed for s.c. injected VV [22]. *In vivo* MVA tropism and, in particular, its capacity to infect MΦs and DCs is of crucial importance since both these cells function as professional APCs and could be involved in antigen presentation and in subsequent T cell priming.

Among APC subsets, MΦs have been somewhat neglected because few reports produce evidence that these particular cells have any decisive function besides their phagocytic activity. While an immunostimulatory role in the generation of cytotoxic T lymphocytes was observed, their exact contribution to this antiviral activity remains unknown [31]. Intriguingly, we report here that Mos/MΦs flowed massively into ADLNs after MVA i.d. inoculation. Previous work has noted the massive presence of VV-infected MΦs in DLNs, but has pointed out only the role of infected DCs in direct VV antigen presentation to CD8^+^ T cells [22], [23]. Our data demonstrate that MΦs have a previously neglected role – the triggering of T cell priming after i.d. antigen delivery. Although less effective than DCs, MΦs are capable of activating naive T lymphocytes [32]. Here, the contribution of MΦs in T cell priming after MVA i.d. inoculation may be related to their local abundance in DLNs and the inflammatory environment [25], [26]. We did not directly examine whether the differences we saw in the immunization routes was caused by direct or cross-presentation. Due to its ability to infect and to efficiently produce viral and recombinant antigens in both professional APC and non-professional APC, MVA has characteristics that enable direct priming as well as cross priming. A previous report suggested that the MVA–specific CD8 T cell response strongly though not exclusively depends on cross priming [33]. As the IFN-γ RT response as a proportion of the overall MVA response (Figure S2) was lower, it suggests that the increased overall MVA response may be due to cross-priming.

Both dermal DCs and CD8α^+^ DCs are widely involved in the presentation of viral antigens [34], [35], [36], [37], [38], [39]. In VV infection, the conventional CD8α^+^ DCs are the main DC subset that primes the anti-VV CTL response *in vivo* [40]. Recent findings also reveal that pDCs can present VV antigens to CD8-T cells [41]. Unfortunately, because we recovered only small quantities of these pDCs from DLNs, we could not directly test them as APCs in our system. It is tempting, however, to hypothesize that they may have a decisive function, either directly by antigen presentation, or indirectly, by promoting other DCs as suggested by others [42]. Surprisingly, we found that among the DC subsets we tested, only mDCs rather than lymphoid DC efficiently presented MVA antigens to both CD4^+^ and CD8^+^ T cells. Nevertheless, the divergence between our results and those previously published may be due to the experimental system studying polyclonal antigen-responses to MVA. Indeed, our study examined the overall T cell response elicited *in vivo*, whereas most -if not all- previous data come from studies under optimal conditions in a peptide-specific transgenic T cell system and focused on class I-restricted presentation of viral antigens by DCs [22], [23], [40]. Taken together, these results challenge the idea that not only CD8α^+^ DCs play an important part in priming immunity during poxvirus infection [22], [40] but also mDCs could have a crucial role. However, MΦs involvement in T cell priming would depend on the original site of antigen encounter.

Whether the mDCs able to present antigens arise from the peripheral tissues where they have phagocytosed MVA *in situ* remains to be confirmed. The phenotypes of dermis- and muscle-infiltrating cells as well as the phenotype of CD11b^+^CD11c^+^ DCs recovered from ADLNs and IDLNs coincide with that of dermal DCs or DCs differentiated from Mos recruited to the inoculation site [36], [43]. These CD11b^+^CD11c^+^ DCs began increasing in frequency and number at 24 hr after antigen delivery and may therefore correspond to migratory DCs that have traveled from the periphery towards DLNs [36]. These results are in accordance with one antigen presentation scenario proposed by Villadangos and Schnorrer, involving both migratory and resident DC populations. In this scenario, the migratory DCs are themselves infected by the virus, which has no cytopathic effect, and thus present antigens by MHC class I and class II molecules in the DLNs [44]. This scenario is consistent with the phenotype of APCs involved in T cell priming in our model.

The generation of multifunctional CD8^+^ T cells with enhanced effector functions has been demonstrated after MVA vaccination in humans [45]. Among the functions tested, MVA induced mainly polyfunctional CD8^+^ T cells that produced MIP-1β, IFN-γ, and TNF-α. In our mouse model, the relative proportion of multiple cytokine production by CD8 cells was much higher in the i.d. (30%) than the i.m. (13%) group (p<0.05), whereas similar relative proportions of multiple cytokine-producing CD4 cells were found by both routes. After i.d. antigen delivery, single producers of IFN-γ and TNF-α as well as CD8^+^ T cells producing both cytokines were dominant, whereas after i.m. administration, we detected mainly CD8^+^ T cells producing a single cytokine. The induction of multifunctional antigen-specific CD8^+^ T cells, in particular those producing both IFN-γ and TNF-α, may contribute to effective immunity, for T cells with this functional signature persist in individuals vaccinated with VV [46]. This suggests that i.d. antigen delivery might be a better route for the generation and maintenance of multifunctional CD8 T cells.

An important finding is that F4/80^+^ cells isolated from ADLNs are endowed with the potential to modulate the cytokine production of T cells in i.m.-inoculated mice. Antigen presentation by distinct APCs results in different patterns of cytokine production in responding T cells [47]. The involvement of MΦs in T cell priming after i.d. antigen delivery may therefore account for the effect on the immune response of this MVA administration route. Together, these results suggest that the quality of antigen-specific T cells is imprinted early after antigen encounter and might reflect differences in cell tropism, antigen accessibility and presentation.

Overall, these results emphasize the contribution of migratory cells to MVA–induced T cell priming and suggest that the *in vivo* antigen distribution, the mobilization of particular APC subsets toward the DLNs, and their anatomical location may together explain the differences we observed in MVA–elicited immune responses according to the delivery route. We have demonstrated that this antigen delivery route is of crucial importance in driving immunocompetent cells to lymphoid organs where they subsequently induce distinct functional populations of T cells. This study improves our understanding of the mechanisms of antigen delivery and may provide new insights for vaccine development.

## Materials and Methods

### Mice and immunizations

Female C57BL/6 mice (6–8 weeks old) were obtained from Charles River Laboratories (L'Arbresle, France) and were housed under specific pathogen-free conditions. All animals were handled in strict accordance with good animal practice and complied with local animal experimentation and ethics committee guidelines. The study was approved by the “Centre d'experimentation fonctionnelle” review board with authorization number A75-13-08 (CERFA n°50-4341; CERFA n°50-4342). For all experiments, mice were immunized i.m. (in the anterior tibialis) or i.d. (in the back of the ear) with 5.10^6^ Plaque Forming Units (PFU) of MVA WT (MVA) strain, a recombinant strain of MVA expressing HIV-1-Reverse Transcriptase (rMVA–RT), or recombinant strain of MVA expressing the enhanced GFP (rMVA–*egfp*), which was kindly produced by Dr B.Verrier from a strain originally provided by Transgene Laboratories (Strasbourg, France).

### Interferon-γ ELISPOT assay

The IFN-γ ELISPOT kit (Diaclone, Strasbourg, France) was used under the manufacturer's suggested conditions. Briefly, DLN cells (1.0×10^5^ cells) were harvested at day 7 after MVA immunization. Cells were incubated with MVA (0.1 PFU/cell) or overlapping HIV-1-RT 15 mer-peptides for 36 hr at 37°C on a plate coated with purified anti-IFN-γ. After incubation, the plates were incubated with biotinylated anti- IFN-γ mAb for 90 min., then by streptavidin-alkaline phosphatase conjugated for 1 hour and BCIP/NBT substrate buffer. The frequency of spot-forming cells (SFC) was measured with an automated microscope (Zeiss).

### MVA–egfp neutralization assay

Neutralizing activities were evaluated by an assay based on flow cytometric detection of GFP as previously described [48]. A detailed description of the method used is included Figure S1.

### Preparation of cell suspensions

Ears, muscles, and lymph nodes (LN) cells were dissociated from the matrix by a 45-minute incubation at 37°C with 400 units/ml collagenase (Sigma-Aldrich) in RPMI 1640 medium (Invitrogen). Cells were then filtered through a 70-µm nylon mesh, and counted before Ab staining.

### Flow cytometry

Conjugated Abs against CD11b-FITC or -APC (clone M1/70), Ly-6G-PE (clone 1A8), Ly-6C-biotin (clone AL-21), CD11c-PE or -APC (clone HL3), CD8α (Ly-2)–PercP-Cy5 (clone 53-6.7), CD45R/B220-PE (clone RA3-6B2) were purchased from BD Biosciences, and anti-F4/80 (clone A3-1)-PE was purchased from Serotec (Oxford, UK). After a 20-min incubation with purified anti-CD16/CD32 mAb (clone 2.4G2, BD Biosciences), surface staining was performed in PBS-FACS (PBS, 5% FCS, 0.05% sodium azide). Abs were incubated for 20 min and then washed twice in PBS-FACS. When biotinylated Abs were used, the secondary reagent was streptavidin-PercP (BD Biosciences). Fluorescence was analyzed on a total of 2.10^4^ cells of each population per sample with a FACSCalibur (BD Biosciences) and FlowJo software (Treestar, Inc., San Carlos, CA).

### Intracellular cytokine staining

For intracytoplasmic cytokine detection, cell suspensions were stimulated overnight with MVA (0,1 PFU/cell) and then incubated for 4 hr at 37°C with brefeldin A at 5 µg/ml. The cells were stained with CD4-PercP (clone RM4-5) or CD8α-PercP (clone 53-6.7). After fixation and permeabilization with 1X PBS-2% FCS-0.1% saponin, cells were stained with APC-conjugated anti-mouse IFN-γ, FITC-conjugated anti-IL-2, and PE-conjugated anti- TNF-α (BD Biosciences). Cells were then run on a FACSCalibur (BD Biosciences, San Jose, CA): 50,000 live CD8 or CD4 positive events per sample were analyzed with FlowJo software (Treestar, Inc., San Carlos, CA). Boolean combination gating was performed to determine the frequencies of expression profiles corresponding to the seven different combinations of cytokines.

### Isolation of DCs and MΦs from lymph nodes and activation of naive T cells

DLNs from ten mice per group were digested with collagenase, filtered through a 70-µm nylon mesh, and layered on a Histodenz gradient (Sigma-Aldrich, St. Louis, MO) prepared in RPMI 10% FCS. Low-density cells were collected and washed in PBS-FCS. DCs were enriched by positive immunomagnetic selection that used anti-CD11c-coated beads according to the manufacturer's recommendations (Miltenyi Biotec, Bergisch Gladbach, Germany). The MΦs among the CD11c^−^ cells were further selected with F4/80-PE Ab and anti-PE coated magnetic beads (MACs, Miltenyi Biotec). For DC subset isolation, DLNs cells were Ab-depleted by a mixture of anti-CD3ε (145-C11), anti-B220 (RA3-6B2), anti-NK1.1 (NKR-P1B and NKR-P1C), and anti-Ly-6G (1A8) mAbs all PE-conjugated. The Ab-coated cells were removed with anti-PE-coupled magnetic beads. DCs were further enriched by positive selection of DCs with CD11c beads (Miltenyi Biotec), labeled with CD8α-PE-Cy7 (Ly-2) and CD11b-FITC (M1/70), and sorted by a FACSVantage instrument cell sorter (BD Biosciences) to complete purification of the DCs into subsets. To isolate T cell populations, the high density cells recovered after Histodenz gradient on naive mice LNs were enriched by positive immunomagnetic selection that used anti-CD4-coated or anti-CD8 beads (Miltenyi Biotec, Bergisch Gladbach, Germany). Analysis of the sorted populations showed purities ranging from 91 to 97%.

For ex-vivo APC assays, nitrocellulose 96-well plates (Millipore) were coated as described above. MACS-isolated CD4^+^ or CD8^+^ T cells obtained from naive LNs were plated at 10^6^ cells/well. The indicated numbers of sorted DC populations (total DCs, MΦs, myeloid DCs, CD8〈^+^ DCs) from DLNs on day 2 after i.d. and i.m. MVA inoculation were added to the wells in 100-µl aliquots. After 36 hr of incubation at 37°C, the plates were treated and SFC frequencies determined as described above.

### Adoptive transfer of F4/80^+^ cells

DLNs taken on day 2 from ten mice inoculated i.d. with MVA or from control mice were treated with collagenase, filtered through a 70-mm nylon mesh and layered on a Histodenz gradient. After low-density cells were collected and washed in PBS-FCS, F4/80^+^ cells were isolated by using F4/80-PE and anti-PE coated magnetic microbeads (MACs, Miltenyi Biotec). 5.10^5^ F4/80 positive cells were injected i.v. into i.m.-MVA inoculated mice on day 2. At day 7, the DLN T cell cytokine profile was determined by intracellular cytokine staining.

### In vivo confocal microscopy

*In vivo* biodistribution of rMVA–e*gfp* was monitored with noninvasive fiber-based confocal microscopy (CellVizio®, Mauna Kea Technologies) as previously described [24]. The S-1500 optical probe used for acquisition of the images presented here has a diameter of 1.5 mm providing images immediately below the surface of biological tissue, with a slice thickness of 15 µm and a lateral resolution of 5 µm. Fluorescent spots in 10 different areas per organ were collected for at least 3 mice (n = 30). Saline buffer-treated mouse tissue was used for background fluorescence detection.

### Immunofluorescence on tissue sections

Sections cut from frozen DLNs were incubated with rat anti-CD45R/B220, biotinylated, rabbit-anti-vaccinia (AbCys s.a.), purified rat anti-F4/80, or purified hamster anti-CD3ε antibodies. Then, slides were incubated with secondary antibody goat anti-rat IgG Alexa 488, or 633, anti-hamster-Alexa 594, Alexa Fluor®488 or Texas red conjugated-streptavidin (Probes, Invitrogen). Slides were mounted with Fluoromount-G (Southern Biotechnology Associates, Birmingham, AL) and analyzed under an Olympus fluorescence microscope. Images were acquired with a Evolution VF/Qimaging digital camera and Qimaging QCapture Pro software. Images were then processed with Adobe Photoshop (Adobe Systems, San Jose, CA). For confocal microscopy, we used the Leica Sp2 AOBS confocal microscope and processed the images with Leica imagej64 software.

### Statistical analysis

Data are presented as the mean±standard deviation. Statistical analyses compared the groups of mice with the unpaired *t*-test and Graphpad software. P values of less than 0.05 were considered statistically significant.

## Supporting Information

Figure S1 Time course of antibody response to MVA immunization. Groups of mice (six per group) were immunized i.d. (filled circle) or i.m. (filled square) with 5.106 PFU of MVA. Sera were collected at different time points following immunization and neutralizing antibody titers were determined using the neutralization assay that measures the reduction in infectivity of rMVA-egfp. This assay was performed by adding 2.5×10^4^ PFU of rMVA-egfp to 40 µl of serial dilution of heat-inactivated serum. The plate was incubated for 1 hr at 37°C. Then, 1×10^5^ HeLa cells were added to 50 µl of culture and incubated for 16 hr at 37°C. GFP expression was analyzed on a total of 10,000 live events per sample with a FACSCalibur and CellQuestPro software (BD Biosciences). The percentage of neutralization was defined as ratio of the reduction in the number of GFP-expressing cells to the number of GFP-expressing cells in untreated control wells and calculated as follows: (1−[percentage of GFP-expressing cells/percentage of GFP-expressing cells in untreated controls])×100. The serum dilution that reduced the percentage of GFP-expressing cells by 50% (IC50) was determined by nonlinear regression with the PRISM software package (version 4.00; GraphPad Software, Inc., San Diego, CA). Geometric mean titres are shown. Differences between i.d. and i.m. immunized groups were analyzed using unpaired t-test, **P<0.01.
(4.80 MB TIF)

Figure S2 Cellular immune response to a foreign gene expressed by a recombinant strain of MVA expressing HIV-1-Reverse Transcriptase (rMVA-RT). Groups of mice were immunized i.d. or i.m. with 5.106 PFU of rMVA-RT or saline buffer as a control. Seven days following vaccination, mice were killed, and DLNs were harvested, and stimulated with overlapping HIV-1-RT 15 mers peptides in vitro. IFN-γ-producing T cells were evaluated by ELISPOT assay. Representative data from two independent experiments are presented as the mean±standard deviation (n = 11 individual mice). * (P<0.05) represents the differences between mock-injected mice and MVA-inoculated mice, (NS, Non Significant). The statistical differences values between i.d. and i.m.-MVA inoculated groups of mice are indicated.
(1.85 MB TIF)

Figure S3 Time course of MVA-specific T cell response in the DLNs. Groups of mice were immunized i.d. or i.m. with 5.106 pfu of MVA or saline buffer as a control. Seven, fourteen, thirty, and sixty days following vaccination, mice were killed, and DLNs were harvested. IFN-γ-producing T cells were evaluated by ELISPOT assay. Representative data from two (days 30 and 60) or three (days 7 and 14) independent experiments are presented as the mean±standard deviation (n = 16 individual mice for day 7 and day 14, n = 6 individual mice for day 30 and 60). ** (P<0.01) *** (P<0.001) represent the differences between between i.d. and i.m. MVA-immunized groups.
(2.37 MB TIF)

Figure S4 Time course of MVA-specific T cell response in spleen. (A) Groups of mice were immunized i.d. or i.m. with 5.106 pfu of MVA or saline buffer as a control. Seven, fourteen, thirty, and sixty days following vaccination, mice were killed, and spleens were harvested. IFN-γ-producing T cells were evaluated by ELISPOT assay. Representative data from two (days 30 and 60) or three (days 7 and 14) independent experiments are presented as the mean±standard deviation (n = 16 individual mice for day 7 and day 14, n = 6 individual mice for day 30 and 60). * (P<0.05) ** (P<0.01) represent the differences between between i.d. and i.m. MVA-immunized groups. (B) MVA-specific T-cell responses in the DLNs on day 7 were measured with an intracellular cytokine staining assay. The magnitude of MVA-specific CD4+ T cells producing cytokines is shown. (C) The functional composition of the CD4+ T cell response is presented. The pie charts present the mean frequencies of the CD4+ T cells positive for the indicated cytokines. TP, triple producers; DP, double producers; and SP, single producers. (D) The magnitude of the total CD8+ T cell response is presented. (E) The cytokine coexpression profile of MVA-specific CD8+ T cells is shown (as in C). Data are representative of two (B,C,D,E) independent experiments (n = 10 individual mice for each group).
(0.95 MB EPS)

Figure S5 Isolation and phenotype of purified myeloid APCs. (A) Dot-plots representing purified CD11c+ DCs and CD11c− F4/80+ MΦs. 80% of the isolated DCs were also positive for the F4/80 marker. (B) Phenotypic analysis of the DLNs CD11b+CD11c+ DCs 48 hours after MVA injection. CD11b+CD11c+ cells were analyzed by flow cytometry for the expression of MHC class II, Ly-6C, and F4/80. The percentage of cells with fluorescence intensity over control staining is indicated.
(1.46 MB EPS)

Figure S6 Myeloid APCs quantitatively and qualitatively shape MVA-specific T-cell responses in the spleen. (A) F4/80+ cells were isolated from MVA i.d. inoculated mice on day 2 or from control mice, and transferred intravenously into i.m. MVA-inoculated mice the same day. Seven days after inoculation, IFN-γ-producing splenic T cells were quantified by ELISPOT assay. Representative data from two independent experiments are presented (n = 10 individual mice for each group). * p<0.05, NS Non Significant. (B) Splenic cells were isolated from recipient mice on day 7 to determine the cytokine coexpression profile of T cells. Cells were stimulated in vitro for 12 h with MVA antigen. Cell surfaces were stained with anti-CD8α and anti-CD4, and then cells were intracellularly stained with anti-IFN-γ, anti-TNF-α, and anti-IL-2. Cytokine co-expression patterns are represented as the mean frequencies of the T-cell subsets positive for the indicated cytokines. Representative data from two independent experiments are presented (n = 10 individual mice for each group).
(0.95 MB EPS)

## References

[1] Belyakov IM, Earl P, Dzutsev A, Kuznetsov VA, Lemon M (2003). Shared modes of protection against poxvirus infection by attenuated and conventional smallpox vaccine viruses.. Proc Natl Acad Sci U S A.

[2] Drexler I, Staib C, Kastenmuller W, Stevanovic S, Schmidt B (2003). Identification of vaccinia virus epitope-specific HLA-A*0201-restricted T cells and comparative analysis of smallpox vaccines.. Proc Natl Acad Sci U S A.

[3] Earl PL, Americo JL, Wyatt LS, Eller LA, Whitbeck JC (2004). Immunogenicity of a highly attenuated MVA smallpox vaccine and protection against monkeypox.. Nature.

[4] Wyatt LS, Earl PL, Eller LA, Moss B (2004). Highly attenuated smallpox vaccine protects mice with and without immune deficiencies against pathogenic vaccinia virus challenge.. Proc Natl Acad Sci U S A.

[5] Meseda CA, Garcia AD, Kumar A, Mayer AE, Manischewitz J (2005). Enhanced immunogenicity and protective effect conferred by vaccination with combinations of modified vaccinia virus Ankara and licensed smallpox vaccine Dryvax in a mouse model.. Virology.

[6] Phelps AL, Gates AJ, Hillier M, Eastaugh L, Ulaeto DO (2007). Comparative efficacy of modified vaccinia Ankara (MVA) as a potential replacement smallpox vaccine.. Vaccine.

[7] Carroll MW, Moss B (1997). Host range and cytopathogenicity of the highly attenuated MVA strain of vaccinia virus: propagation and generation of recombinant viruses in a nonhuman mammalian cell line.. Virology.

[8] Drexler I, Heller K, Wahren B, Erfle V, Sutter G (1998). Highly attenuated modified vaccinia virus Ankara replicates in baby hamster kidney cells, a potential host for virus propagation, but not in various human transformed and primary cells.. J Gen Virol.

[9] Blanchard TJ, Alcami A, Andrea P, Smith GL (1998). Modified vaccinia virus Ankara undergoes limited replication in human cells and lacks several immunomodulatory proteins: implications for use as a human vaccine.. J Gen Virol.

[10] Stittelaar KJ, Kuiken T, de Swart RL, van Amerongen G, Vos HW (2001). Safety of modified vaccinia virus Ankara (MVA) in immune-suppressed macaques.. Vaccine.

[11] Puissant B, Combadiere B (2006). Keeping the memory of smallpox virus.. Cell Mol Life Sci.

[12] Gomez CE, Najera JL, Krupa M, Esteban M (2008). The poxvirus vectors MVA and NYVAC as gene delivery systems for vaccination against infectious diseases and cancer.. Curr Gene Ther.

[13] Combadiere B, Boissonnas A, Carcelain G, Lefranc E, Samri A (2004). Distinct time effects of vaccination on long-term proliferative and IFN-gamma-producing T cell memory to smallpox in humans.. J Exp Med.

[14] Amara RR, Nigam P, Sharma S, Liu J, Bostik V (2004). Long-lived poxvirus immunity, robust CD4 help, and better persistence of CD4 than CD8 T cells.. J Virol.

[15] Miller JD, van der Most RG, Akondy RS, Glidewell JT, Albott S (2008). Human effector and memory CD8+ T cell responses to smallpox and yellow fever vaccines.. Immunity.

[16] Seder RA, Darrah PA, Roederer M (2008). T cell quality in memory and protection: implications for vaccine design.. Nat Rev Immunol.

[17] Estcourt MJ, Letourneau S, McMichael AJ, Hanke T (2005). Vaccine route, dose and type of delivery vector determine patterns of primary CD8+ T cell responses.. Eur J Immunol.

[18] Shen X, Wong SB, Buck CB, Zhang J, Siliciano RF (2002). Direct priming and cross-priming contribute differentially to the induction of CD8+ CTL following exposure to vaccinia virus via different routes.. J Immunol.

[19] Tscharke DC, Karupiah G, Zhou J, Palmore T, Irvine KR (2005). Identification of poxvirus CD8+ T cell determinants to enable rational design and characterization of smallpox vaccines.. J Exp Med.

[20] Sanchez-Puig JM, Sanchez L, Roy G, Blasco R (2004). Susceptibility of different leukocyte cell types to Vaccinia virus infection.. Virol J.

[21] Chahroudi A, Chavan R, Kozyr N, Waller EK, Silvestri G (2005). Vaccinia virus tropism for primary hematolymphoid cells is determined by restricted expression of a unique virus receptor.. J Virol.

[22] Norbury CC, Malide D, Gibbs JS, Bennink JR, Yewdell JW (2002). Visualizing priming of virus-specific CD8+ T cells by infected dendritic cells in vivo.. Nat Immunol.

[23] Hickman HD, Takeda K, Skon CN, Murray FR, Hensley SE (2008). Direct priming of antiviral CD8+ T cells in the peripheral interfollicular region of lymph nodes.. Nat Immunol.

[24] Iga M, Boissonnas A, Mahe B, Bonduelle O, Combadiere C (2007). Single CX3CL1-Ig DNA administration enhances T cell priming in vivo.. Vaccine.

[25] Hamilton-Easton A, Eichelberger M (1995). Virus-specific antigen presentation by different subsets of cells from lung and mediastinal lymph node tissues of influenza virus-infected mice.. J Virol.

[26] Usherwood EJ, Hogg TL, Woodland DL (1999). Enumeration of antigen-presenting cells in mice infected with Sendai virus.. J Immunol.

[27] Pulendran B (2004). Modulating vaccine responses with dendritic cells and Toll-like receptors.. Immunol Rev.

[28] Gomez CE, Najera JL, Domingo-Gil E, Ochoa-Callejero L, Gonzalez-Aseguinolaza G (2007). Virus distribution of the attenuated MVA and NYVAC poxvirus strains in mice.. J Gen Virol.

[29] Abadie V, Badell E, Douillard P, Ensergueix D, Leenen PJ (2005). Neutrophils rapidly migrate via lymphatics after Mycobacterium bovis BCG intradermal vaccination and shuttle live bacilli to the draining lymph nodes.. Blood.

[30] Bonneau M, Epardaud M, Payot F, Niborski V, Thoulouze MI (2006). Migratory monocytes and granulocytes are major lymphatic carriers of Salmonella from tissue to draining lymph node.. J Leukoc Biol.

[31] Karupiah G, Buller RM, Van Rooijen N, Duarte CJ, Chen J (1996). Different roles for CD4+ and CD8+ T lymphocytes and macrophage subsets in the control of a generalized virus infection.. J Virol.

[32] Pozzi LA, Maciaszek JW, Rock KL (2005). Both dendritic cells and macrophages can stimulate naive CD8 T cells in vivo to proliferate, develop effector function, and differentiate into memory cells.. J Immunol.

[33] Gasteiger G, Kastenmuller W, Ljapoci R, Sutter G, Drexler I (2007). Cross-priming of cytotoxic T cells dictates antigen requisites for modified vaccinia virus Ankara vector vaccines.. J Virol.

[34] Allan RS, Smith CM, Belz GT, van Lint AL, Wakim LM (2003). Epidermal viral immunity induced by CD8alpha+ dendritic cells but not by Langerhans cells.. Science.

[35] Zhao X, Deak E, Soderberg K, Linehan M, Spezzano D (2003). Vaginal submucosal dendritic cells, but not Langerhans cells, induce protective Th1 responses to herpes simplex virus-2.. J Exp Med.

[36] Itano AA, McSorley SJ, Reinhardt RL, Ehst BD, Ingulli E (2003). Distinct dendritic cell populations sequentially present antigen to CD4 T cells and stimulate different aspects of cell-mediated immunity.. Immunity.

[37] Allan RS, Waithman J, Bedoui S, Jones CM, Villadangos JA (2006). Migratory dendritic cells transfer antigen to a lymph node-resident dendritic cell population for efficient CTL priming.. Immunity.

[38] Mount AM, Smith CM, Kupresanin F, Stoermer K, Heath WR (2008). Multiple dendritic cell populations activate CD4+ T cells after viral stimulation.. PLoS ONE.

[39] Bedoui S, Whitney PG, Waithman J, Eidsmo L, Wakim L (2009). Cross-presentation of viral and self antigens by skin-derived CD103+ dendritic cells.. Nat Immunol.

[40] Belz GT, Smith CM, Eichner D, Shortman K, Karupiah G (2004). Cutting edge: conventional CD8 alpha+ dendritic cells are generally involved in priming CTL immunity to viruses.. J Immunol.

[41] Bontkes HJ, Ruizendaal JJ, Schreurs MW, Kramer D, Meijer CJ (2005). Antigen gene transfer to human plasmacytoid dendritic cells using recombinant adenovirus and vaccinia virus vectors.. Cell Oncol.

[42] Yoneyama H, Matsuno K, Toda E, Nishiwaki T, Matsuo N (2005). Plasmacytoid DCs help lymph node DCs to induce anti-HSV CTLs.. J Exp Med.

[43] Leon B, Lopez-Bravo M, Ardavin C (2007). Monocyte-derived dendritic cells formed at the infection site control the induction of protective T helper 1 responses against Leishmania.. Immunity.

[44] Villadangos JA, Schnorrer P (2007). Intrinsic and cooperative antigen-presenting functions of dendritic-cell subsets in vivo.. Nat Rev Immunol.

[45] Precopio ML, Betts MR, Parrino J, Price DA, Gostick E (2007). Immunization with vaccinia virus induces polyfunctional and phenotypically distinctive CD8(+) T cell responses.. J Exp Med.

[46] Hammarlund E, Lewis MW, Hansen SG, Strelow LI, Nelson JA (2003). Duration of antiviral immunity after smallpox vaccination.. Nat Med.

[47] Macatonia SE, Hsieh CS, Murphy KM, O'Garra A (1993). Dendritic cells and macrophages are required for Th1 development of CD4+ T cells from alpha beta TCR transgenic mice: IL-12 substitution for macrophages to stimulate IFN-gamma production is IFN-gamma-dependent.. Int Immunol.

[48] Cosma A, Buhler S, Nagaraj R, Staib C, Hammarin AL (2004). Neutralization assay using a modified vaccinia virus Ankara vector expressing the green fluorescent protein is a high-throughput method to monitor the humoral immune response against vaccinia virus.. Clin Diagn Lab Immunol.

