# Supplementary figures and images for: Original Encounter with Antigen Determines Antigen-Presenting Cell Imprinting of the Quality of the Immune Response in Mice
Source: PLoS One. 2009 Dec 7;4(12):e8159. doi: 10.1371/journal.pone.0008159 (PMC2785484; doi:10.1371/journal.pone.0008159)

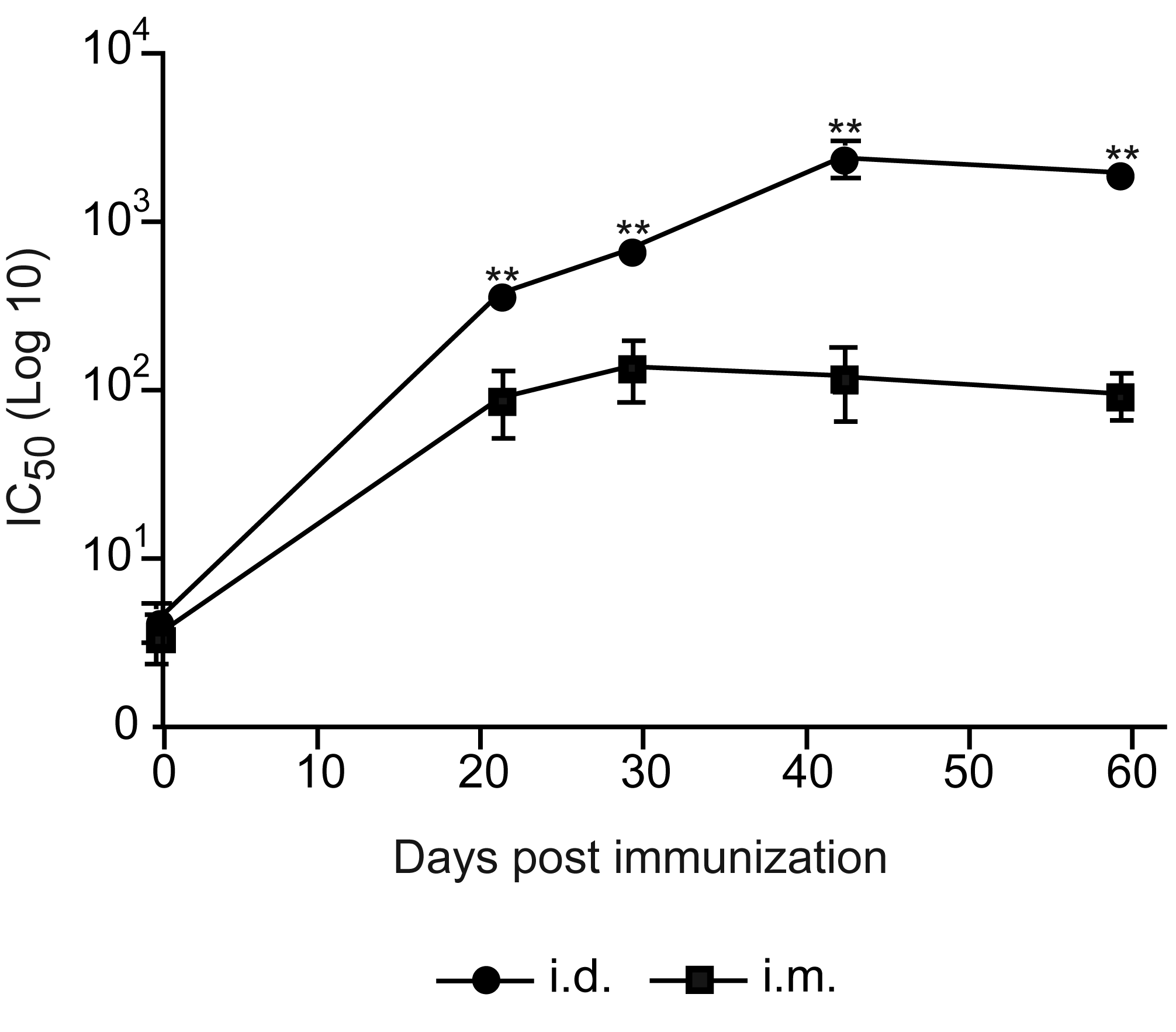

Supplement: Figure S1 — Time course of antibody response to MVA immunization. Groups of mice (six per group) were immunized i.d. (filled circle) or i.m. (filled square) with 5.106 PFU of MVA. Sera were collected at different time points following immunization and neutralizing antibody titers were determined using the neutralization assay that measures the reduction in infectivity of rMVA-egfp. This assay was performed by adding 2.5×10^4^ PFU of rMVA-egfp to 40 µl of serial dilution of heat-inactivated serum. The plate was incubated for 1 hr at 37°C. Then, 1×10^5^ HeLa cells were added to 50 µl of culture and incubated for 16 hr at 37°C. GFP expression was analyzed on a total of 10,000 live events per sample with a FACSCalibur and CellQuestPro software (BD Biosciences). The percentage of neutralization was defined as ratio of the reduction in the number of GFP-expressing cells to the number of GFP-expressing cells in untreated control wells and calculated as follows: (1−[percentage of GFP-expressing cells/percentage of GFP-expressing cells in untreated controls])×100. The serum dilution that reduced the percentage of GFP-expressing cells by 50% (IC50) was determined by nonlinear regression with the PRISM software package (version 4.00; GraphPad Software, Inc., San Diego, CA). Geometric mean titres are shown. Differences between i.d. and i.m. immunized groups were analyzed using unpaired t-test, **P<0.01. (4.80 MB TIF) [file pone.0008159.s001.tif]

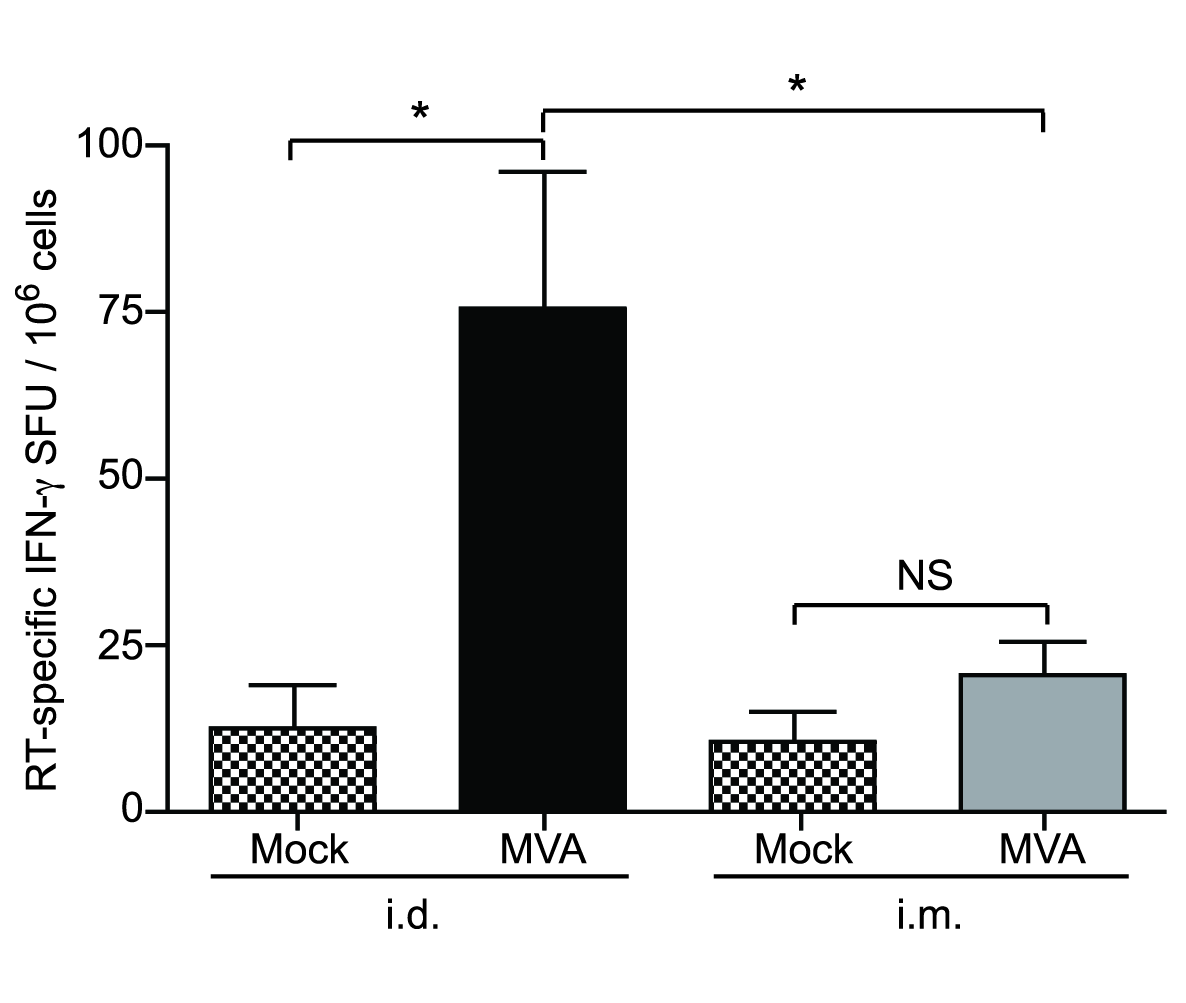

Supplement: Figure S2 — Cellular immune response to a foreign gene expressed by a recombinant strain of MVA expressing HIV-1-Reverse Transcriptase (rMVA-RT). Groups of mice were immunized i.d. or i.m. with 5.106 PFU of rMVA-RT or saline buffer as a control. Seven days following vaccination, mice were killed, and DLNs were harvested, and stimulated with overlapping HIV-1-RT 15 mers peptides in vitro. IFN-γ-producing T cells were evaluated by ELISPOT assay. Representative data from two independent experiments are presented as the mean±standard deviation (n = 11 individual mice). * (P<0.05) represents the differences between mock-injected mice and MVA-inoculated mice, (NS, Non Significant). The statistical differences values between i.d. and i.m.-MVA inoculated groups of mice are indicated. (1.85 MB TIF) [file pone.0008159.s002.tif]

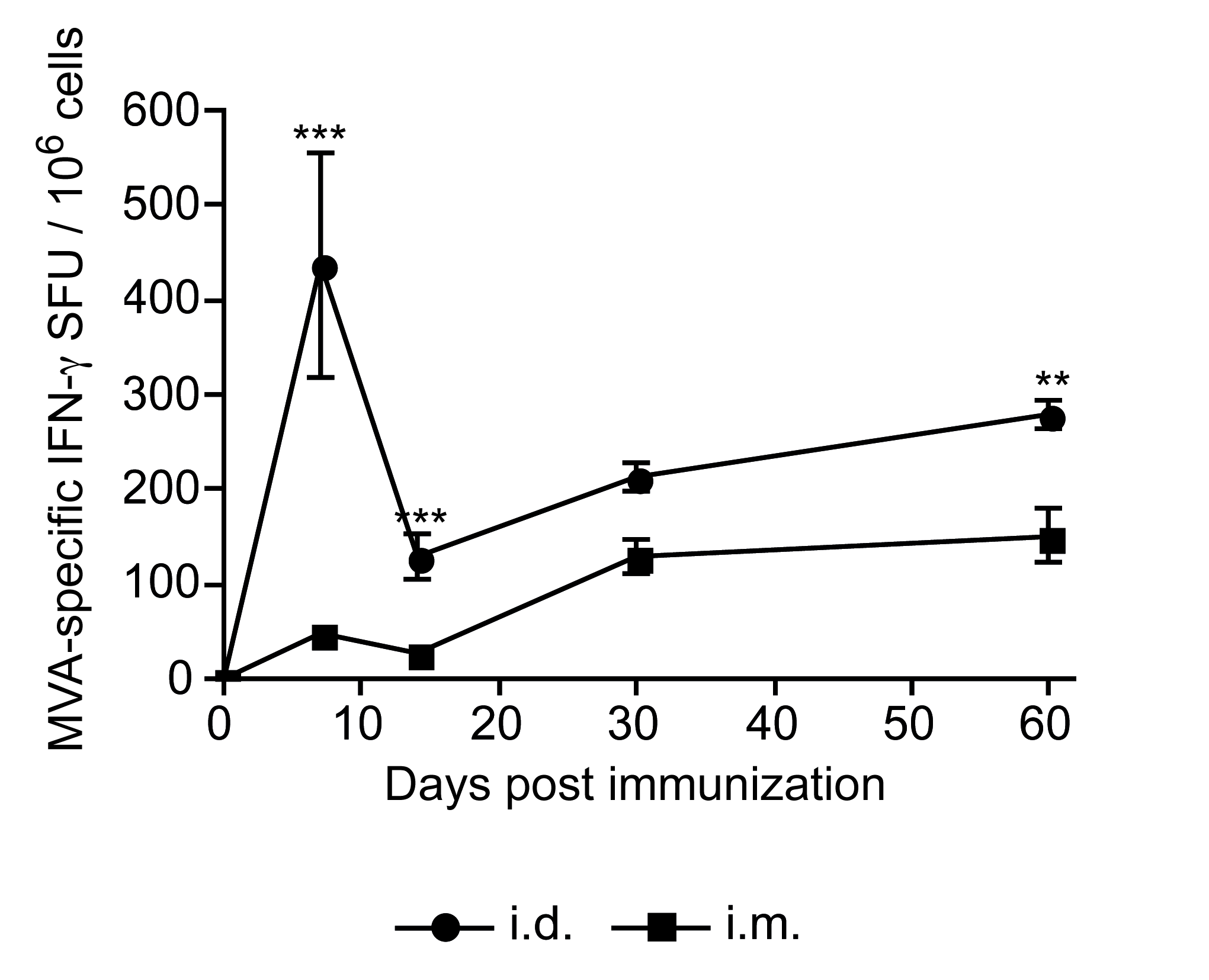

Supplement: Figure S3 — Time course of MVA-specific T cell response in the DLNs. Groups of mice were immunized i.d. or i.m. with 5.106 pfu of MVA or saline buffer as a control. Seven, fourteen, thirty, and sixty days following vaccination, mice were killed, and DLNs were harvested. IFN-γ-producing T cells were evaluated by ELISPOT assay. Representative data from two (days 30 and 60) or three (days 7 and 14) independent experiments are presented as the mean±standard deviation (n = 16 individual mice for day 7 and day 14, n = 6 individual mice for day 30 and 60). ** (P<0.01) *** (P<0.001) represent the differences between between i.d. and i.m. MVA-immunized groups. (2.37 MB TIF) [file pone.0008159.s003.tif]
